# Systematic Review of Newborn Screening Programmes for Spinal Muscular Atrophy

**DOI:** 10.3390/ijns10030049

**Published:** 2024-07-15

**Authors:** Katy Cooper, Gamze Nalbant, Anthea Sutton, Sue Harnan, Praveen Thokala, Jim Chilcott, Alisdair McNeill, Alice Bessey

**Affiliations:** School of Medicine and Population Health, University of Sheffield, Sheffield S1 4DA, UK; g.nalbant@sheffield.ac.uk (G.N.); a.sutton@sheffield.ac.uk (A.S.); s.harnan@sheffield.ac.uk (S.H.); p.thokala@sheffield.ac.uk (P.T.); j.b.chilcott@sheffield.ac.uk (J.C.); a.mcneill@sheffield.ac.uk (A.M.); a.r.bessey@sheffield.ac.uk (A.B.)

**Keywords:** systematic review, spinal muscular atrophy, neonatal screening, newborn screening, SMA birth prevalence, screening program, laboratory methods, *SMN1* and *SMN2* copy numbers

## Abstract

Spinal muscular atrophy (SMA) is a genetic neuromuscular disorder causing the degeneration of motor neurons in the spinal cord. Recent studies suggest greater effectiveness of treatment in the presymptomatic stage. This systematic review synthesises findings from 37 studies (and 3 overviews) of newborn screening for SMA published up to November 2023 across 17 countries to understand the methodologies used; test accuracy performance; and timing, logistics and feasibility of screening. All studies screened for the homozygous deletion of *SMN1* exon 7. Most (28 studies) used RT-PCR as the initial test on dried blood spots (DBSs), while nine studies also reported second-tier tests on DBSs for screen-positive cases. Babies testing positive on DBSs were referred for confirmatory testing via a range of methods. Observed SMA birth prevalence ranged from 1 in 4000 to 1 in 20,000. Most studies reported no false-negative or false-positive cases (therefore had a sensitivity and specificity of 100%). Five studies reported either one or two false-negative cases each (total of six cases; three compound heterozygotes and three due to system errors), although some false-negatives may have been missed due to lack of follow-up of negative results. Eleven studies reported false-positive cases, some being heterozygous carriers or potentially related to heparin use. Time to testing and treatment varied between studies. In conclusion, several countries have implemented newborn screening for SMA in the last 5 years using a variety of methods. Implementation considerations include processes for timely initial and confirmatory testing, partnerships between screening and neuromuscular centres, and timely treatment initiation.

## 1. Introduction

Spinal muscular atrophy (SMA) is an autosomal recessive disease associated with the progressive and irreversible degeneration of lower motor neurons in the anterior horn of the spinal cord and brainstem. The onset of neuromuscular weakness ranges from birth to adulthood. Historically, SMA was classified into discrete types based on age of onset of weakness, with SMA type 0 presenting neonatally and type 4 in early adulthood. It is now apparent that SMA spans a continuum without discrete subtypes. The vast majority of cases of SMA (95%) are due to a homozygous deletion of exons 7 and 8 of *SMN1* [1]. A minority are compound heterozygotes, where one copy of *SMN1* is deleted and the other has a missense variant. Overall, these genetic changes lead to a decrease in functional SMN protein and ultimately lead to patients developing SMA. The related *SMN2* gene can also make SMN protein, but only around 10% of the SMN protein from the *SMN2* gene is functional. Therefore, *SMN2* can partially compensate for deletions or mutations in *SMN1*. People can have multiple copies of the *SMN2* gene with a higher number of *SMN2* copies generally correlating with reduced disease severity [2]. However, it is not currently possible to accurately predict severity from genetic information alone.

Many countries have begun to introduce newborn screening for SMA. Newborn screening aims to identify babies with SMA via the screening of all newborns in a country or area. Newborn screening for SMA often uses real-time quantitative polymerase chain reaction (qRT-PCR) techniques to assess the patient’s *SMN* genes, using DNA isolated from dried blood spots (DBSs) collected soon after birth. Most newborn screening for SMA screens for homozygous deletion of the *SMN1* gene.

Treatments for SMA include nusinersen (Spinraza) [3], an antisense oligonucleotide designed to modify the product of the *SMN2* gene to produce more functional SMN protein, risdiplam (Evrysdi), a small molecule drug that targets the *SMN2* gene to produce more SMN protein [4], and onasemnogene abeparvovec (Zolgensma), a gene therapy which expresses the SMN protein [5]. Recently, treatment of SMA in the presymptomatic stage has been suggested to improve outcomes compared to the treatment of symptomatic disease [6]. Presymptomatic treatment may be facilitated by identifying babies at an early stage via newborn screening [7].

We therefore undertook a systematic review of cohort studies of newborn screening for SMA worldwide to understand the methodologies used and the ability of screening to reliably identify neonates with SMA in the presymptomatic stage.

## 2. Review Methods

### 2.1. Aims of Review

This systematic review aimed to synthesise findings from cohort studies of newborn screening for SMA worldwide to understand the methodologies used; the numbers and potential causes of false-negative and false-positive cases; the test accuracy of screening; and findings relating to the timing, logistics and feasibility of screening. Our systematic review followed the PRISMA guidelines. Our review protocol was registered on PROSPERO (registration number CRD42023473172).

### 2.2. Search Strategy

Searches of MEDLINE, Embase and the Cochrane Library were conducted in November 2023 and covered all dates up to this point. Thesaurus and free-text terms for SMA (plus synonyms) were combined with terms for newborn screening. The search strategy is provided in Appendix A. Recent reviews and relevant studies were also checked, and experts consulted, to identify any additional studies.

### 2.3. Inclusion and Exclusion Criteria

The review included studies of newborn screening for 5q SMA worldwide. Prospective cohort studies and RCTs were eligible for inclusion, while case-control studies were not included. However, a systematic search for case-control studies was undertaken, and a list is provided in Appendix B (Table A1) for information. Studies of both pilot and routine screening were eligible. Relevant outcomes included the observed birth prevalence of SMA; numbers and potential causes of false-negative and false-positive cases; test accuracy outcomes (sensitivity, specificity, positive and negative predictive value); and findings relating to the timing, logistics and feasibility of screening. This review focusses on screening processes and diagnostic follow-up, and it does not seek to evaluate ongoing patient management, patient outcomes or loss to follow-up.

### 2.4. Study Selection and Data Extraction

References were checked for inclusion by one reviewer, and a 10% sample was checked by a second reviewer early in the process to check for consistency in inclusion decisions. Data for all studies were extracted by one reviewer and checked by another. Data were extracted relating to the country/area, whether pilot or routine screening, dates of screening, methodologies for initial and confirmatory testing, and outcomes as listed above.

### 2.5. Risk of Bias Assessment

Risk of bias within included studies was assessed using the Quality Assessment of Diagnostic Accuracy Studies 2 (QUADAS-2) tool [8].

### 2.6. Calculation of Outcome Measures

Test accuracy outcomes were reported as stated in included studies or calculated by the review team where data permitted. As an overview of test accuracy outcomes, true-positive (TP) cases are those who test positive and truly have the condition; true-negative (TN) cases are those who test negative and truly do not have the condition; false-positive (FP) cases are those who test positive but do not have the condition; and false-negative (FN) cases are those who test negative but do actually have the condition. From these numbers, the following test accuracy outcomes were calculated. The positive predictive value is the number of patients correctly testing positive as a percentage of all those with a positive initial test result (TP/[TP+FP]). The negative predictive value is the number of patients correctly testing negative as a percentage of all those with a negative initial test result (TN/[TN+FN]). Sensitivity is the number of patients correctly testing positive as a percentage of all those who truly have the condition (TP/[TP+FN]). Specificity is the number of patients correctly testing negative as a percentage of all those who truly do not have the condition (TN/[TN+FP]).

The aim of most screening programmes was to detect homozygous deletions of *SMN1*. Most screening methods were not designed to identify compound heterozygotes of *SMN1* (around 2–5% of SMA cases). Therefore, sensitivity was calculated in two ways: firstly for detecting homozygous *SMN1* deletions and secondly for detecting any SMA case (including both homozygous deletions and compound heterozygotes; this latter measure would be expected to be a maximum of 95–98%, since compound heterozygotes would not be identified).

In addition, some studies reported conducting “second-tier” (and sometimes “third-tier”) testing on the original DBS when the initial screening result was positive or inconclusive. These additional tests on the original DBS were considered part of the index test when calculating test accuracy outcomes. Conversely, the confirmatory test on a new blood sample, generally conducted in a specialist centre, was considered the reference standard test.

### 2.7. Synthesis Methods

Findings were synthesised via tabulation and narrative synthesis.

## 3. Results

### 3.1. Volume, Type and Setting of Included Studies

**Figure 1:**
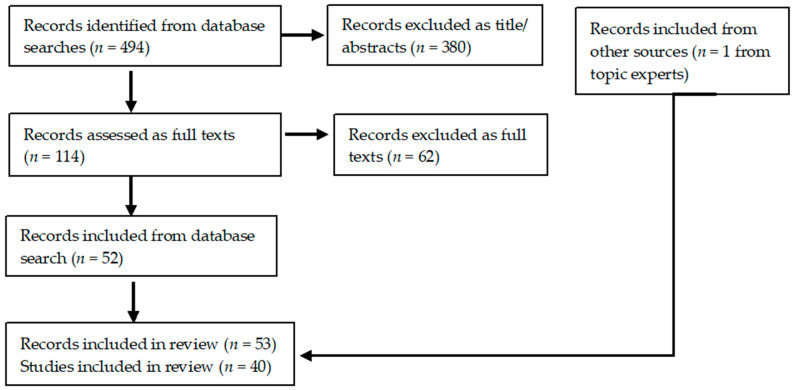
PRISMA flow chart.

The search generated 494 references from the database search and 1 from other sources. In total, 40 studies were included (within 53 references; Table 1). A PRISMA flow diagram is shown in Figure 1.

The review identified 37 cohort studies of newborn screening for SMA [9,10,11,12,13,14,15,16,17,18,19,20,21,22,23,24,25,26,27,28,29,30,31,32,33,34,35,36,37,38,39,40,41,42,43,44,45,46,47,48,49,50,51,52,53,54,55,56,57]. No RCTs of newborn screening were identified. Of the 37 cohort studies, 34 studies reported prospective screening programmes of newborns using DBS screening, while three studies reported analyses using cohorts of anonymised DBS samples (one in Ohio [44], two in China [54,55]). Of the 34 prospective screening studies, 22 were pilot studies, 9 were routine screening, and 3 were both. In terms of location, four studies reported nationwide screening (in Germany [17], Latvia [21], Norway [25] and Japan [50]), while 29 covered a particular area or state (and one did not report this [22]). The majority of included references were published between 2019 and 2024, reflecting the recent nature of published studies.

Cohort studies relating to newborn screening programmes for SMA were identified from the following 17 countries (Table 1): the UK [9], Belgium [10,11,12], Germany [13,14,15,16,17,18], Italy [19,20], Latvia [21], Portugal [22], Poland [23], Ukraine [24], Norway [25], Australia [26,27,28,29], the USA, Canada [30,31,32], Brazil [45], Japan [46,47,48,49,50], Taiwan [51,52], China [53,54,55] and Russia [56,57]. The USA screening programmes were reported for several US states: California [33], Georgia [34], Kentucky [35], Massachusetts [36,37], New York [38,39,40], North Carolina [41], Wisconsin [42], Utah [43] and Ohio [44].

In addition, we identified three overviews of screening studies across broader geographical locations (one global, one USA-based and one Canada-based); these overviews reported data on prevalence, screening methodologies and diagnostic accuracy, and they were therefore includable in our review [58,59,60,61]. The global overview published in 2021 suggested that by 2025, newborn screening for SMA was forecast to include 24% of newborns in countries where a disease-modifying drug is available and 8.5% of newborns in countries with no disease-modifying drugs [58]. An overview for Canada reported that SMA newborn screening was available in five of eight Canadian provinces and all three territories by October 2022, and that the number of Canadian newborns screened for SMA increased from 60% in June 2022 to 72% in January 2023 [59]. A similar overview for the USA reported that SMA newborn screening was available in 48 of 53 US states or territories as of December 2022 [60,61].

**Table 1:** Methodologies of screening for SMA.

Study, Location	Duration (Dates)	Pilot or Routine	Area or Nationwide	Index Test: Method	Index Test: 2nd Tier (S+)	Index Test: Type	Index Test: Multiplex?	Confirmatory Test at SC (S+)	*SMN2* Copy No Test (S+)	*N* SMA Cases	*N* Screened	Prevalence
Overviews of geographical areas												
Global overview [58]	Various	Various	Various	Various	Various	Various	Various	Various	Various	288	3,674,277	1 in 12,758
USA overview (29 states) [60,61]	Prevalence data for 2018–2020	Various	Various	Various	Various	Various	Various	Various	Various	219	3,185,560	1 in 14,546
Canada overview [59]	Various	Various	Various	qPCR; MLPA; MassArray	Various	Various	SMA+SCID	MLPA	Various	-	-	-
Prospective screening cohort studies												
UK (Thames Valley) [9]	8 mo (dates NR)	Pilot	Area	-	-	-	SMA only	-	-	-	5691	-
Belgium (Southern) [10,11,12]	3 yr (March 2018 to February 2021)	Pilot	Area	RT-qPCR	Repeat PCR x2 then MLPA	Own test	SMA only	MLPA	MLPA (DBS) + seq (new sample)	10	136,339	1 in 13,634
Germany (Bavaria + NRW) [13,14,15,16,17]	2 yr (January 2018 to January 2020)	Pilot	Area	qPCR	-	Own test	SMA only	MLPA	MLPA (new sample)	43	297,163	1 in 6910
Germany (nationwide) [17]	6 mo (October 2021 to March 2022)	Routine	Nationwide	qPCR	-	-	SMA only	Y (lab discretion)	Lab discretion (new sample)	-	-	1 in 8554
Germany (Heidelberg) [18]	9 mo (July 2021 to March 2022)	Pilot then routine	Area	qPCR	Repeat PCR	Own test	SMA, SCID, SCD	Y (method NR)	Y (method NR; new sample)	14	96,015	1 in 6857
Italy (Lazio and Tuscany) [19]	2 yr (September 2019 to September 2021)	Pilot	Area	RT-qPCR	Repeat PCR	Own test	SMA only	RFLP-PCR + splicing variants	Semi-quant qPCR (new sample)	15	90,885	1 in 6059
Italy (Liguria) [20]	1 yr (NR dates)	Pilot	Area	RT-PCR	-	-	SMA+SCID	MLPA	-	2	8434	1 in 4217
Latvia [21]	10 mo (February 2021 to Nov 2021)	Pilot	Nationwide	qPCR	Repeat PCR	-	SMA only	qPCR + MLPA	MLPA (new sample)	2	10,411	1 in 5205
Portugal [22]	-	Pilot	-	RT-PCR	-	Commercial	-	Y (method NR)	Y (method NR; new sample)	2	25,000	1 in 12,500
Poland (13 districts) [23]	1 yr (from April 2021)	Routine	Area	PCR-HRM	PCR-RFLP or MLPA	Commercial	-	MLPA	-	21	140,000	1 in 6667
Ukraine (near Kyiv) [24]	7 mo (October 2022 to May 2023)	Pilot	Area	-	-	-	-	-	-	11	65,880	1 in 5989
Norway (nationwide) [25]	19 mo (September 2021 to April 2023)	Routine	Nationwide	qPCR	-	-	SMA+SCID	ddPCR then whole-gen seq. If het del: check point mutation	ddPCR, then whole-gen seq (NR location)	10	-	-
Australia (NSW + ACT) [26,27,28]	2.5 yr (August 2018 to January 2021)	Pilot	Area	RT-PCR 4-plex	-	Commercial	SMA+SCID	MLPA	ddPCR + qPCR (new sample)	23	252,081	1 in 10,960
Australia (Queensland) [29]	2 wk (in March 2021)	Pilot	Area	Next-gen seq	-	Commercial	SMA only	MLPA	-	0	2552	-
Canada (Ontario) [30,31]	1 yr (from January 2020)	Pilot then routine	Area	PCR (MassArray)	MLPA	Own test	SMA, SCID hearing	Y (method NR)	MLPA (DBS); Y (method NR; new sample)	5	139,800	1 in 27,960
Canada (Alberta) [32]	1 yr (February 2022 to February 2023)	Pilot	Area	qPCR	Repeat PCR x2	-	SMA+SCID	MLPA	MLPA (new sample)	5	47,005	1 in 9401
USA (California) [33]	18 mo (June 2020 to December 2021)	Routine	Area	RT-PCR	Repeat PCR + ddPCR	-	SMA+SCID	Multiplex PCR	ddPCR (DBS); PCR (new sample)	34	628,791	1 in 18,494
USA (Georgia) [34]	2 yr (February 2019 to February 2021)	Pilot then routine	Area	RT-PCR	-	-	SMA+SCID	Y (method NR)	Y (method NR; new sample)	16	301,418	1 in 18,839
USA (Kentucky) [35]	2 yr (August 2019 to July 2021)	Routine	Area	-	-	-	SMA+SCID	Y (method NR)	Y (method NR; new sample)	11	108,511	1 in 9865
USA (Massachusetts) [36,37]	3 yr (January 2018 to January 2021)	Routine	Area	RT-qPCR	Tier 2: exon 7 variant. Tier 3: sequencing	Own test	SMA+SCID	Y (method NR)	Sequencing (DBS); Y (method NR; new sample)	9	179,467	1 in 19,941
USA (New York State) [38,39]	3 yr (October 2018 to September 2021)	Routine	Area	RT-qPCR	Repeat PCR	Commercial	SMA+SCID	Y (method NR)	qPCR + ddPCR (DBS); Y (method NR; new sample)	34	Nearly 650,000	1 in 19,118
USA (3 hospitals New York City) [40]	1 yr (January 2016 to January 2017)	Pilot	Area	RT-qPCR	Repeat PCR	Commercial	SMA+SCID	Y (method NR)	Y (method NR; new sample)	1	3826	1 in 3826
USA (North Carolina) [41]	26 mo (October 2018 to December 2020)	Pilot	Area	RT-qPCR	Repeat PCR	Commercial	SMA only	ddPCR or MLPA-seq	Y (method NR; new sample)	1	12,065	1 in 12,065
USA (Wisconsin) [42]	1 yr (October 2019 to October 2020)	Routine	Area	Multiplex RT-PCR	ddPCR on new DBS punch	Own test	SMA+SCID	Y (method NR)	ddPCR (DBS); Y (method NR; new sample)	6	60,984	1 in 10,164
USA (Utah) [43]	5 yr (2018 to 2023)	Routine	Area	-	-	-	-	-	-	13	239,844	1 in 18,450
Brazil (Sao Paulo + Rio Grande do Sul) [45]	NR	Pilot	Area	RT-qPCR	-	Commercial	SMA only	MLPA	MLPA (NR location)	4	40,000	1 in 10,000
Japan (Kumamoto) [46]	1 yr (February 2021 to January 2022)	Pilot	Area	RT-PCR	-	Commercial	-	qPCR + MLPA	MLPA (NR location)	1	13,587	1 in 13,587
Japan (Osaka) [47,48]	8 mo (February 2021 to September 2021)	Pilot	Area	RT-qPCR	-	-	SMA, SCID, BCD	MLPA	MLPA (new sample)	0	22,951	-
Japan (Hyogo) [49]	18 mo (February 2021 to August 2022)	Pilot	Area	RT-qPCR	Repeat PCR	Commercial	-	MLPA + ddPCR	ddPCR (new sample)	2	8336	1 in 4168
Japan (49 hosp, 23 prefectures) [50]	15 mo (January 2018 to April 2019)	Pilot	Nationwide	PCR then RT-mCOP-PCR	PCR-RFLP	Own test	-	Y (method NR)	-	0	4157	-
Taiwan (University Hospital) [51,52]	5 yr (November 2014 to December 2019)	Pilot	Area	RT-PCR	ddPCR	-	SMA+SCID	MLPA	ddPCR (DBS) + MLPA (new sample)	21	364,000	1 in 17,333
China (6 hospitals) [53]	4 mo (March 2018 to June 2018)	Pilot	Area	DNA mass spectrometry	-	Own test	-	MLPA	MLPA (NR location)	3	29,364	1 in 9788
Russia (Moscow) [56]	2.5 yr (August 2019 to January 2022)	Pilot	Area	PCR melting curve	PCR-RFLP	Commercial	SMA only	MLPA + Sanger sequencing	MLPA (new sample)	3	23,405	1 in 7801
Russia (Saint Petersburg) [57]	11 mo (January 2022 to November 2022)	Pilot	Area	RT-PCR	Repeat PCR on new DBS punch	Commercial	SMA only	Different RT-PCR + MLPA	Y (method + location NR)	4	36,140	1 in 9035
Studies using anonymised DBS samples												
USA (Ohio) [44]	N/A	Anonymised samples	N/A	PCR	Competitive PCR	Own test	SMA only	N/A	Y (method NR; DBS)	-	40,103	1 in 10,026
China (Hunan province) [54]	N/A	Anonymised samples	N/A	RT-PCR	-	Own test	SMA only	N/A	-	-	753	-
China (southwest) [55]	N/A	Anonymised samples	N/A	RT-PCR	Repeat PCR + DNA seq	Own test	SMA only	N/A	-	-	2000	-

Abbreviations: ACT, Australian Capital Territory; BCD, B-cell deficiency; DBS, dried blood spot; ddPCR, droplet digital PCR; het del, heterozygous deletion; HRM, high-resolution melting; mCOP-PCR, modified competitive oligonucleotide priming-PCR; MLPA, multiplex ligation-dependent probe amplification; mo, months; next-gen seq, next-generation sequencing; N, number; NSW, New South Wales; NR, not reported; NRW, North Rhine-Westphalia; PCR, polymerase chain reaction; qPCR, quantitative polymerase chain reaction; RFLP, restriction fragment length polymorphism; RT-PCR, real-time polymerase chain reaction; S+, screen positives; SC, specialist centre; SCD, sickle cell disease; SCID, severe combined immunodeficiency; seq, sequencing; SMA, spinal muscular atrophy; *SMN1/2*, survival motor neuron 1/2; whole-gen seq, whole-genome sequencing; yr, year.

### 3.2. Prevalence of SMA from Newborn Screening Studies

The total number of newborns screened per study (across the 34 prospective studies) ranged from 2552 to 650,000 (Table 1). The number of identified SMA cases ranged from 0 to 43. Based on these data, the observed prevalence of SMA ranged from around 1 in 4000 to 1 in 20,000 (Table 1). It is possible that some SMA cases were not detected via screening, firstly because most screening programmes are not designed to identify compound heterozygotes (2–5% of SMA cases) and secondly because some false-negative cases may have been missed if they were not diagnosed clinically within the study timeframe. This could mean that prevalence is underestimated in some studies.

### 3.3. Methodologies of Screening for SMA

#### 3.3.1. Aims of Screening

Details of screening methods are shown in Table 1. All studies aimed to screen for the homozygous deletion of *SMN1* exon 7 so would not identify compound heterozygotes. However, some studies also identified heterozygous carriers, including the New York State pilot study [40], a study in Norway [25], a study in Russia [57] and a study in China using anonymised DBS samples [54]. In New York State [40], parents of heterozygous carriers were offered genetic testing to determine whether both parents were carriers. In the Norwegian study [25], babies with a heterozygous deletion were further tested for a specific point mutation, so compound heterozygotes with this mutation would have been identified.

#### 3.3.2. Methodologies for Initial Screening of DBS Sample

In terms of screening methods, the majority of studies (*n* = 28 of 37) used real-time PCR (RT-PCR) or quantitative PCR (qPCR) on the DBS sample as a first-tier screening method. Other studies used high-resolution melting PCR (*n* = 2, Poland [23] and Russia [56]), modified competitive oligonucleotide priming-PCR (mCOP-PCR, *n* = 1, Japan [50]), DNA mass spectrometry (*n* = 1, China [53]), next-generation sequencing (*n* = 1, Australia [29]), or did not report the method (*n* = 4).

SMA screening was reported to be multiplexed with screening for severe combined immunodeficiency (SCID) in around 40% of studies (15 of 37), including studies in the USA, Canada, Australia, Germany, Italy, Norway, Japan and Taiwan. In addition, a few studies reported multiplex screening with other conditions, including SCID plus sickle cell disease (Germany [18]); SCID plus B-cell deficiency (Japan [47]) or SCID plus hearing loss (Canada [30]). Table 1 also notes whether programmes used their own lab-developed test or a commercial test; this varied between studies but was often unclear from the study report.

In all studies, screen-negative cases were not followed up further. Screen-positive cases could undergo three types of further testing, as described below: (i) second-tier testing for *SMN1* deletion on the original DBS; (ii) referral to a specialist centre for confirmatory testing of *SMN1* deletion on a fresh blood sample; and (iii) testing for *SMN2* copy number.

#### 3.3.3. Methodologies for Second-Tier Testing of DBS Sample

Here, we refer to “second-tier testing” as any further testing for *SMN1* deletion on the original DBS for screen-positive cases. Some but not all studies included second-tier tests. In total, 12 studies reported repeating the initial PCR on screen-positive cases. Furthermore, nine studies conducted other types of second-tier test on the original DBS for screen-positive cases, including droplet digital PCR (ddPCR, *n* = 3) [33,42,51], multiplex ligation-dependent probe amplification (MLPA; *n* = 3) [10,23,30], restriction fragment length polymorphism PCR (RFLP-PCR; *n* = 3) [23,50,56], and one study with three-tier testing in screen positives (Massachusetts: PCR, then testing for exon 7 variants, then sequencing [36]). These second-tier tests on the DBS were considered part of the index test rather than the reference standard within this review when determining test accuracy.

#### 3.3.4. Methodologies for Confirmatory Testing in a Specialist Centre

Babies who were screen positive following DBS testing were generally referred to a specialist centre for consultation, and a fresh blood sample was taken for confirmatory testing for *SMN1* deletion. This confirmatory testing, rather than the various tiers of screening on the initial DBS, was considered the reference standard within this review when determining test accuracy. Methods of confirmatory testing included the following (some studies used more than one method): MLPA (*n* = 17) [10,13,20,21,23,26,29,32,41,45,46,47,49,52,53,56,57], PCR (*n* = 5) [21,33,44,46,57], ddPCR (*n* = 3) [25,41,49], sequencing (*n* = 3) [25,55,56], restriction fragment length polymorphism PCR (RFLP-PCR, *n* = 1) [19], analysis of splicing variants (*n* = 1) [19], or the method was not reported (*n* = 15). The three studies which used cohorts of anonymised DBS samples [44,54,55] could not conduct confirmatory testing on a new blood sample and relied instead on the second-tier testing of screen-positive cases using the original DBS samples.

#### 3.3.5. Methodologies of Testing for *SMN2* Copy Number

Screen-positive cases also generally underwent testing for *SMN2* copy number. This was most commonly conducted on a new blood sample in the specialist centre, but it was also conducted on the DBSs in some studies (Table 1). Again, a variety of methods were reported for this, including the following (some studies used more than one method): MLPA (*n* = 11) [10,13,21,30,32,45,46,47,52,53,56], ddPCR (*n* = 7) [25,26,33,38,42,49,52], qPCR (*n* = 4) [19,26,33,38], sequencing (*n* = 3) [10,25,36], or the method was not reported (*n* = 18).

### 3.4. Test Accuracy Outcomes from Screening Studies

#### 3.4.1. Overview of Test Accuracy Data

Most cohort studies reported the total number of newborns screened, the number testing positive, and the number of true-positive and false-positive cases. Confirmatory testing on a new blood sample was only performed on babies who tested positive in the initial screen. Therefore, false-negative cases (those missed by screening) were generally only identified if they later presented with symptoms, and so numbers of false-negative cases may have been underestimated, particularly later-onset cases of SMA which may not be clinically apparent in early life. Some studies did not mention false-negative cases at all, so it was unclear whether information on missed cases had actually been sought.

The numbers of false-positive and false-negative cases, and associated test accuracy outcomes, are summarised in Table 2.

#### 3.4.2. Positive Predictive Value

It was generally possible to calculate the positive predictive value; however, this was based on small numbers of cases. Due to the low prevalence, a small number of false-positives could substantially reduce the positive predictive value. Where this could be calculated, it was 100% in 15 studies [10,13,18,19,21,30,33,38,40,42,44,46,53,56,57], and in the remainder, it was 4% [55], 17% [49], 38% [34], 50% [41], 69% [35], 80% [45], 83% [32], 90% [36], 92% [17], 93% [43] and 95% [26]. A lower positive predictive value means that a study had more false-positives. As noted earlier, second-tier and third-tier tests on the original DBS were considered part of the index test when calculating test accuracy outcomes, while confirmatory testing on a new blood sample in a specialist centre was considered the reference standard. If only the first-tier test was considered to be the index test, the positive predictive value would be lower, as some false-positives are ruled out during subsequent tiers of testing on the DBS. Possible reasons for false-positives are discussed below and summarised in Table 3.

#### 3.4.3. Negative Predictive Value

The negative predictive value could generally be calculated, but it may be overestimated due to the underestimation of false-negative cases, as described above. Where the negative predictive value could be calculated, it was 100% in all studies (to the nearest whole percentage point). This was the case even where a study reported some false-negatives due to the low prevalence of SMA in the population.

#### 3.4.4. Sensitivity

It was generally possible to calculate sensitivity, but again, this may be overestimated due to the underestimation of false-negative cases. Also, due to the low prevalence, a small number of false-negatives could substantially reduce the sensitivity. As noted in the Methods, sensitivity was calculated in two ways: firstly for detecting homozygous *SMN1* deletions (which were the target of screening), and secondly for detecting any SMA case (including compound heterozygotes which could not be identified via screening). Sensitivity for detecting homozygous *SMN1* deletions (where calculable) was 100% in 23 studies, and it was 91% and 94% in two further studies with two and one false-negative cases, respectively [26,34]. In addition, three studies each identified one compound heterozygous case (identified via symptoms and classed as false-negative); the sensitivity for these studies, calculated for all SMA cases rather than just homozygous deletions, was 90%, 95% and 98% [10,17,51].

#### 3.4.5. Specificity

Specificity could generally be calculated, because the number of false-positive cases was generally reported. Where specificity could be calculated, it was 100% in all studies (to the nearest whole percentage point). This was the case even where a study reported some false-positives due to the low prevalence of SMA in the population.

### 3.5. False-Negatives, False-Positives, Incomplete Results and Incidental Findings

Details and possible causes of false-positive and false-negative cases, as well as initial incomplete results and incidental findings, are provided in Table 3.

#### 3.5.1. False-Negative Cases

The majority of studies did not report any false-negative cases. Only six false-negative cases were reported across five studies [10,17,26,34,52]; these babies were generally identified when they presented with symptoms. Three false-negative babies were found to be compound heterozygotes, which cannot be identified via screening for homozygous deletions of *SMN1* [10,17,52]. Three further false-negative cases were related to system or human errors [26,34] (Table 3).

#### 3.5.2. False-Positive Cases

The majority of studies (eighteen studies) did not report any false-positive cases, while six studies reported one false-positive each [26,32,36,41,43,45], and one study each reported 4 false-positives [17], 5 false-positives [35], 10 false-positives [49], 22 false-positives [55] or 24 false-positives [34] (Table 3; the remaining studies did not report this information). False-positives were identified upon confirmatory testing on a new blood sample. Some false-positives were found to be heterozygous carriers of the *SMN1* deletion [17,32,45], or had sequence variants in the *SMN1* or *SMN2* genes [26], or recombination between the genes [52]. Some babies with false-positive results were unwell in hospital at the time of sample collection [34], or premature [34], or also had a false-positive SCID screen [35]; the correlation between these factors and a false-positive result was unclear. Some false-positive cases were suggested to be due to heparinised and/or diluted blood in the DBS sample [49] (Table 3).

#### 3.5.3. Initial Incomplete Results

Thirteen studies reported cases with incomplete or uncertain results on the initial test, who then had a definitive result on further tiers of testing [19,21,32,33,34,36,40,41,44,45,47,52,56] (these were not classed as false-positives since the issues were resolved through further testing of the initial DBS sample, which was considered to be part of the index test process). Some were thought to be due to the use of heparin [19]; some related to babies in the neonatal intensive care unit (NICU), possibly due to presence of a PCR inhibitor [36]; some were due to poor DNA quality or quantity [21,33,40,41,44,45,47,52]; some were due to system or handling errors [32]; and some were not explained further (Table 3).

#### 3.5.4. Incidental Findings, Sibling Diagnosis and Sequence Variants

Four studies reported cases of siblings being diagnosed with SMA following a positive screening case [10,13,19,56], and one study reported the identification of an unrelated blood disorder [41], while two studies reported initial uncertain results relating to variants of uncertain significance in *SMN1* exon 7 [36,40] (further details in Table 3).

### 3.6. Risk of Bias in Included Studies

Risk of bias in the included studies is shown in Table 4. The included studies were assessed using the QUADAS-2 quality assessment tool, which was tailored to the review question.

In terms of patient selection, 37 of 40 studies were considered to have a low risk of bias due to being cohort studies including a consecutive or random sample of patients (Table 3). Regarding the index test, 39 of 40 studies were considered to have a low risk of bias since the index tests were interpreted without knowledge of the reference standard and did not require the consideration of different thresholds. Furthermore, all the included studies had low concern for applicability for patient selection, index test and reference standard domains, apart from one study [54] being unclear in the reference standard domain.

However, all studies (*n* = 40) were considered to have a high risk of bias for the “reference standard” and “flow and timing” domains, because screen-negative patients did not undergo confirmatory testing, and the results of the index test were likely to have been known when interpreting the reference standard.

### 3.7. Timing of Testing Process

Some studies noted timings of the testing process; timings from birth are reported in Table 5. Median time from birth to DBS sampling was generally 1–6 days, and median time from birth to DBS receipt at the screening centre was generally 2–6 days (or 75 days in one study). Median time from birth to initial screening results ranged from 3 to 18 days. Median time from birth to specialist consultation ranged from 5 to 33 days, while confirmatory results on a new blood sample were available at a median age of 11–28 days. Treatment start was more variable, as it was reported as occurring at a median age of 15–48 days (or 106 days in one study).

Some studies reported the point at which parents were contacted. This was often on the same day as, or soon after, the positive screening result with a specialist appointment arranged for soon after this for examination and confirmatory blood test.

### 3.8. Workflow and Consent Processes

Table 6 summarises information on workflow and consent processes. In terms of workflow, studies varied widely in terms of volume of samples processed, which ranged from 300 per week to 2000 per day. Some screening programmes used opt-in processes and some used opt-out processes. Where reported, consent rates were generally high (over 90%), and this increased when SMA became part of routine screening.

### 3.9. Organisational Considerations, Implementation and Barriers

Some studies reported on organisational and implementation issues and barriers or delays to treatment, as summarised in Table 7.

The most commonly cited barriers leading to delayed treatment were related to (a) testing, e.g., requirement to obtain confirmatory testing results prior to application for treatment; (b) medical issues, e.g., SMA-related or other health issues; (c) financial issues, e.g., problems with insurance authorisation or reimbursement of treatment; and (d) logistical issues, e.g., delayed arrival of the samples at the lab due to problems with transportation, and transporting patients to the centre for confirmatory testing and treatment.

Included studies highlighted some points to be considered before SMA newborn screening is implemented as routine screening at the national or regional level. These included (a) beginning with a pilot project; (b) establishing a well-thought-out implementation process, including developing the screening assay, staffing, selection of specialist centres, funding, regulatory requirements, and process for follow-up care and presymptomatic treatment; (c) logistical considerations, e.g., operation of screening laboratories on weekends, reduction in time to transport samples from the collection site to screening laboratories, and time required for confirmatory testing and treatment approval; and (d) establishing partnerships between newborn screening staff and neuromuscular specialists and patient organisations to reduce delays and promote family-centred care.

Additional ongoing uncertainties included treatment cost-effectiveness and reimbursement; uncertainty regarding long-term outcomes for presymptomatic patients; and uncertainties about management of patients with ≥4 *SMN2* copies.

## 4. Discussion

This review identified 34 prospective cohort studies (plus three overviews and three cohort analyses of anonymised DBSs) evaluating pilot or routine newborn screening for SMA across 17 countries. All studies screened for homozygous deletion of *SMN1* exon 7. Most (28 of 37) used RT-PCR to detect homozygous *SMN1* deletion, and nine studies included additional second-tier tests on dried blood spots (DBSs) for screen-positive cases. Around 40% multiplexed SMA screening with screening for severe combined immunodeficiency (SCID). Babies testing positive via DBSs were referred for confirmatory testing on a new blood sample via MLPA, RT-PCR, ddPCR, RFLP-PCR or sequencing.

Across studies, six false-negative cases were identified via symptoms: three compound heterozygotes and three due to system errors. False-positive cases ranged from *n* = 0 to *n* > 10; some were heterozygous carriers or potentially related to heparin use. The positive predictive value ranged from 4% to 100% depending on the false-positive rate. Sensitivity was 100% in most studies, although some false-negatives may have been missed. The specificity and negative predictive value were close to 100% due to the low prevalence of SMA. Time to testing and treatment varied between studies.

The identification of false-positive cases and initial incomplete results (for example due to heterozygosity for *SMN1* deletion, *SMN* gene sequence variants, gene recombination, presence of PCR inhibitors or issues with DNA quality or quantity) highlights the importance of confirmatory testing. This may include second-tier testing on the initial DBS, which may rule out some false-positive cases without anxiety to families as well as confirmatory testing on a new blood sample. Furthermore, confirmatory testing together with genetic counselling in a clinical setting may ensure the cascade testing of family members, identify family members at risk of developing SMA, and provide information regarding family planning.

The majority of included references were published between 2019 and 2024, reflecting the fact that newborn screening is currently being piloted, evaluated or implemented in several countries worldwide. Previous reviews of newborn screening for SMA [62,63,64,65,66,67] have generally identified smaller numbers of studies due to the volume of articles reported very recently.

Observed prevalence estimates for 5q SMA ranged from 1 in 4000 to 1 in 20,000, which tallies with the reported prevalence of 1 in 6000 to 1 in 30,000 in a recent review [68]. The apparently wide variation in estimates may be due to the small numbers of cases identified in the various studies (so, for example, one missed case may change the estimate).

In terms of limitations, some information was not well reported, such as the reasons for inconclusive or false-positive results. The test methods for the various tiers of DBS testing, confirmatory testing, and *SMN2* copy number testing were not always clearly reported, and the review indicates that there is still relatively wide variation in the methods used.

Further research may focus on the most appropriate testing methods for both DBSs and confirmatory testing as well as the potential for adding SMA screening into routine newborn screening processes. Further work on implementation factors may inform how best to facilitate the timely identification and treatment of patients at a presymptomatic or early symptomatic stage. Our review does not seek to evaluate ongoing patient management, patient outcomes or loss to follow-up of screened babies, but such information would be valuable in order to understand whether SMA screening programmes are fulfilling their potential in enabling the early management of babies with SMA. There are also ongoing uncertainties around managing patients with four *SMN2* copies who may not have been diagnosed until much later in life in the absence of screening.

## 5. Conclusions

In the last five years, several countries have evaluated newborn screening for SMA. Across 37 studies, 6 false-negative cases were identified, while false-positive cases per study ranged from 0 to more than 10. Positive predictive value ranged from 4% to 100%; sensitivity was 100% in most studies; while specificity and negative predictive value were close to 100% due to the low prevalence of SMA. Implementation considerations include processes for timely initial and confirmatory testing, partnerships between screening and neuromuscular centres, and timely treatment initiation.

## Figures and Tables

**Table 2:** Test accuracy of screening for SMA.

Study, Location	*N* Screened	*N* Testing Positive	*N* SMA Cases	TP	FP	FN	TN	PPV	NPV	Sensitivity	Specificity
Overviews of geographical areas											
Global overview [58]	3,674,277	307	288	288	19	0	3,673,970	94%	100%	100%	100%
Prospective screening cohort studies											
Belgium (southern) [10,11,12]	136,339	9	10	9	0	1 (comp heteroz)	136,329	100%	100%	100% [homoz del] 90% [all SMA]	100%
Germany (Bavaria + NRW) [13,14,15,16,17]	297,163	43	43	43	0	0	297,120	100%	100%	100%	100%
Germany (nationwide) [17]	-	50	47	46	4	1 (comp heteroz)	-	92%	-	100% [homoz del] 98% [all SMA]	-
Germany (Heidelberg) [18]	96,015	14	14	14	0	0	96,001	100%	100%	100%	100%
Italy (Lazio and Tuscany) [19]	90,885	15	15	15	0	0	90,870	100%	100%	100%	100%
Latvia [21]	10,411	2	2	2	0	0	10,409	100%	100%	100%	100%
Australia (NSW + ACT) [26,27,28]	252,081	22	23	21	1	2	252,057	95%	100%	91%	100%
Australia (Queensland) [29]	2552	0	0	0	0	0	2552	-	100%	-	100%
Canada (Ontario) [30,31]	139,800	5	5	5	0	0	139,795	100%	100%	100%	100%
Canada (Alberta) [32]	47,005	6	5	5	1	0	46,999	83%	100%	100%	100%
USA (California) [33]	628,791	34	34	34	0	0	628,757	100%	100%	100%	100%
USA (Georgia) [34]	301,418	39	16	15	24	1	301,378	38%	100%	94%	100%
USA (Kentucky) [35]	108,511	16	11	11	5	0	108,495	69%	100%	100%	100%
USA (Massachusetts) [36,37]	179,467	10	9	9	1	0	179,457	90%	100%	100%	100%
USA (New York State) [38,39]	Nearly 650,000	34	34	34	0	0	649,966	100%	100%	100%	100%
USA (3 hospitals New York City) [40]	3826	1	1	1	0	0	3825	100%	100%	100%	100%
USA (North Carolina) [41]	12,065	2	1	1	1	0	12,063	50%	100%	100%	100%
USA (Wisconsin) [42]	60,984	6	6	6	0	0	60,978	100%	100%	100%	100%
USA (Utah) [43]	239,844	14	13	13	1	0	239,830	93%	100%	100%	100%
Brazil [45]	40,000	5	4	4	1	0	39,995	80%	100%	100%	100%
Japan (Kumamoto) [46]	13,587	1	1	1	0	0	13,586	100%	100%	100%	100%
Japan (Osaka) [47,48]	22,951	0	0	0	0	0	22,951	-	100%	-	100%
Japan (Hyogo) [49]	8336	12	2	2	10	0	8324	17%	100%	100%	100%
Japan (49 hosp, 23 prefectures) [50]	4157	0	0	0	0	0	4157	-	100%	-	100%
Taiwan (University Hospital [51,52]	364,000	-	21	20	-	1 (comp heteroz)	-	-	-	100% [homoz del] 95% [all SMA]	-
China (6 hospitals) [53]	29,364	3	3	3	0	0	29,361	100%	100%	100%	100%
Russia (Moscow) [56]	23,405	3	3	3	0	0	23,402	100%	100%	100%	100%
Russia (Saint Petersburg) [57]	36,140	4	4	4	0	0	36,136	100%	100%	100%	100%
Studies using anonymised DBS samples											
USA (Ohio) [44]	40,103	4	-	4	0	-	-	100%	-	-	-
China (southwest) [55]	2000	23	-	1	22	-	-	4%	-	-	-

Abbreviations: ACT, Australian Capital Territory; comp heteroz, compound heterozygotes; FN, false-negative; FP, false-positive; homoz del, homozygous deletion; NPV, negative predictive value; NSW, New South Wales; NR, not reported; NRW, North Rhine-Westphalia; PPV, positive predictive value; SMA, spinal muscular atrophy; TN, true negative; TP, true positive.

**Table 3:** False-negatives, false-positives, incomplete results and incidental findings.

Study, Location	Index Test: Method	*N* Screened	False-Negatives	False-Positives	Initial Incomplete Results	Additional Findings
Prospective screening cohort studies						
Belgium (southern) [10,11,12]	Index: RT-qPCR 2nd tier: Repeat PCR x2 then MLPA Confirmatory: MLPA	136,339	FN: *n* = 1: Compound heterozygote	-	-	*n* = 1 case had siblings identified with SMA
Germany (Bavaria + NRW) [13,14,15,16,17]	Index: qPCR; 2nd tier: NR Confirmatory: MLPA	297,163	-	-	-	*n* = 2 cases had siblings identified with SMA
Germany (nationwide) [17]	Index: qPCR; 2nd tier: NR Confirmatory: Y (lab discretion)	NR	FN: *n* = 1: Compound heterozygote	FP: *n* = 4: 1 had two normal copies of *SMN1* 2 heterozygous carriers 1 inconsistent results with different parts of DBS; final result unclear All in first 2 mo; process modified	-	-
Italy (Lazio and Tuscany) [19]	Index: RT-qPCR 2nd tier: Repeat PCR Confirmatory: RFLP-PCR + splicing variants	90,885	-	-	Some early failed tests; reduced by avoiding heparin-coated capillaries Failed samples required manual DNA extraction; all then successfully screened	*n* = 1 case had siblings identified with SMA
Latvia [21]	Index: qPCR 2nd tier: Repeat PCR Confirmatory: qPCR + MLPA	10,411	-	-	*n* = 40 cases required repeat sampling due to poor DNA quality (quality of punch or manual mistakes during DNA isolation)	-
Australia (NSW + ACT) [26,27,28]	Index: RT-PCR 4-plex; 2nd tier: NR Confirmatory: MLPA	252,081	FN: *n* = 2: 1 system error 1 sample not received	FP: *n* = 1: Homozygous for rare variant in SMN1 or SMN2; possible parental consanguinity	-	-
Canada (Alberta) [32]	Index: qPCR 2nd tier: Repeat PCR x2 Confirmatory: MLPA	47,005	-	FP: *n* = 1: Heterozygous carrier. First sample positive (delayed in transit); further tests negative	*n* = 1 sample misplaced, not tested within 10 days, therefore assumed positive at initial screen, later negative	-
USA (California) [33]	Index: RT-PCR 2nd tier: Repeat PCR + ddPCR Confirmatory: Multiplex PCR	628,791	-	-	*n* = 5 required repeat DBS; 2 inconclusive on initial and repeat samples; NR reason for 3. New sample for each; all 5 negative	-
USA (Georgia) [34]	Index: RT-PCR; 2nd tier: NR Confirmatory: Y (method NR)	301,418	FN: *n* = 1: Human error in first month of pilot	FP: *n* = 24 13 in pilot year; reasons NR 9 sick in hospital at sample collection; 3 of these premature	*n* = 147 had inconclusive results (NR what happened to these); 126 of 147 also inconclusive for SCID screening	-
USA (Kentucky) [35]	Index: NR; 2nd tier: NR Confirmatory: Y (method NR)	108,511	-	FP: *n* = 5: Reasons NR; 4/5 also had false-positive SCID screen	-	-
USA (Massachusetts) [36,37]	Index: RT-qPCR 2nd tier: Tier 2: exon 7 variant. Tier 3: sequencing Confirmatory: Y (method NR)	179,467	-	FP: *n* = 1: During first months; sample may have contained inhibitor	Single Tier 1 assay would have had more false-positives More NICU babies required Tier 2/3 screen; may involve PCR inhibitor	*n* = 10 *SMN1* hybrids with exon 7 variant (assumed normal; n = 6 followed, all healthy at 6 mo)
USA (New York State) [38,39]	Index: RT-qPCR 2nd tier: Repeat PCR Confirmatory: Y (method NR)	3826	-	-	3% initial failure (poor DNA quality or quantity; all negative or carriers on retest) *n* = 33 (0.9%) initial equivocal; on retest, *n* = 30 negative, *n* = 2 heterozygous carriers, *n* = 1 retested equivocal (rare sequence variant; significance unknown)	*n* = 1 with variant of unknown significance (see left) Also screened for heterozygous carriers
USA (North Carolina) [41]	Index: RT-qPCR 2nd tier: Repeat PCR Confirmatory: ddPCR or MLPA-seq	12,065	-	FP: *n* = 1: Likely due to unrelated blood disorder (low white blood cell count)	*n* = 2 not tested; insufficient quantity *n* = 36 first test above cut-off; on retest, *n* = 2 positive, *n* = 34 normal	*n* = 1 FP with unrelated blood disorder (low white blood cell count)
USA (Utah) [43]	Index: NR; 2nd tier: NR Confirmatory: NR	239,844	-	FP: *n* = 1: No further detail	-	-
Brazil [45]	Index: RT-qPCR; 2nd tier: NR Confirmatory: MLPA	40,000	-	FP: *n* = 1: Heterozygous carrier	*n* = 11,289 insufficient material for testing at initial screening; NR whether retested	-
Japan (Osaka) [47,48]	Index: RT-qPCR; 2nd tier: NR Confirmatory: MLPA	22,951	-	-	*n* = 265 (1.1%) invalid e.g., insufficient blood volume; excluded with no re-testing	-
Japan (Hyogo) [49]	Index: RT-qPCR; 2nd tier: Repeat PCR Confirmatory: MLPA + ddPCR	8336	-	FP: *n* = 10: May be related to use of heparinized or diluted blood in DBSs	-	-
Taiwan (University Hospital) [51,52]	Index: RT-PCR; 2nd tier: ddPCR Confirmatory: MLPA	364,000	FN: *n* = 1: Compound heterozygote	FP: NR: States primers modified to avoid early first-tier false-positives	*n* = 50 unsatisfactory results; all negative following repeat DNA extraction and RT-PCR	-
Russia (Moscow) [56]	Index: PCR melting curve 2nd tier: PCR-RFLP Confirmatory: MLPA + sequencing	36,140	-	-	*n* = 219 initial ambiguous; analysed with PCR-RFLP; possibly due to 1 copy of SMN1 and multiple copies of SMN2	*n* = 1 sibling identified Identified parents/siblings as carriers
Studies using anonymised DBS samples						
USA (Ohio) [44]	Index: PCR; 2nd tier: Competitive PCR Confirmatory: N/A	40,103	-	-	*n* = 7 (0.02%) required repeat extraction from DBS; all robust result on re-testing	-
China (southwest) [55]	Index: RT-PCR; 2nd tier: PCR + seq Confirmatory: N/A	2000	-	FP: *n* = 22: No further detail	-	-

Abbreviations: ACT, Australian Capital Territory; DBS, dried blood spot; ddPCR, droplet digital PCR; DNA, deoxyribonucleic acid; FN, false-negative; FP, false-positive; MLPA, multiplex ligation-dependent probe amplification; mo, months; NICU, neonatal intensive care unit; NR, not reported; NRW, North Rhine-Westphalia; NSW, New South Wales; PCR, polymerase chain reaction; qPCR, quantitative polymerase chain reaction; RFLP, restriction fragment length polymorphism; RT-PCR, real-time polymerase chain reaction; SCID, severe combined immunodeficiency; seq, sequencing; SMA, spinal muscular atrophy; *SMN1/2*, survival motor neuron 1/2.

**Table 4:** Risk of bias in included studies.

Study, Location	Patient Selection					Index Test				Ref Standard				Flow + Timing			
	Consecutive or Random Sample	Case-Control Design Avoided	Avoided Inappropriate Exclusions	Risk of Bias Overall	Concerns Re Applicability to Question	Interpreted without Knowledge of Ref Standard	If Threshold Used, Was It Pre-Specified (None Required: Y)	Risk of Bias Overall	Concerns re Applicability to Question	Likely to Correctly Classify Condition	Interpreted without Knowledge of Index Test	Risk of Bias Overall	Concerns Re Applicability to Question	Appropriate Interval between Tests (If Condition will Not Change, Score Y)	All Patients Received (Same) Ref Standard	All Patients Included in Analysis	Risk of Bias Overall
Overviews of geographical areas																	
Global overview [58]	Y	Y	Y	Low	Low	Y	Y	Low	Low	S+: Y	N	High	Low	Y	N	Y	High
USA overview (29 states) [60,61]	Y	Y	Y	Low	Low	Y	Y	Low	Low	S+: Y	N	High	Low	Y	N	Y	High
Canada overview [59]	Y	Y	Y	Low	Low	Y	Y	Low	Low	S+: Y	N	High	Low	Y	N	Y	High
Prospective screening cohort studies																	
UK (Thames Valley) [9]	U	Y	U	Uncl	Low	Y	Y	Low	Low	S+: U	N	High	Low	Y	N	Y	High
Belgium (southern) [10,11,12]	Y	Y	Y	Low	Low	Y	Y	Low	Low	S+: Y	N	High	Low	Y	N	Y	High
Germany (Bavaria + NRW) [13,14,15,16,17]	Y	Y	Y	Low	Low	Y	Y	Low	Low	S+: Y	N	High	Low	Y	N	Y	High
Germany (nationwide) [17]	Y	Y	Y	Low	Low	Y	Y	Low	Low	S+: Y	N	High	Low	Y	N	Y	High
Germany (Heidelberg) [18]	Y	Y	Y	Low	Low	Y	Y	Low	Low	S+: Y	N	High	Low	Y	N	Y	High
Italy (Lazio and Tuscany) [19]	Y	Y	Y	Low	Low	Y	Y	Low	Low	S+: Y	N	High	Low	Y	N	Y	High
Italy (Liguria) [20]	Y	Y	Y	Low	Low	Y	Y	Low	Low	S+: Y	N	High	Low	Y	N	Y	High
Latvia [21]	Y	Y	Y	Low	Low	Y	Y	Low	Low	S+: Y	N	High	Low	Y	N	Y	High
Portugal [22]	Y	Y	Y	Low	Low	Y	Y	Low	Low	S+: Y	N	High	Low	Y	N	Y	High
Poland (13 districts) [23]	Y	Y	Y	Low	Low	Y	Y	Low	Low	S+: Y	N	High	Low	Y	N	Y	High
Ukraine (near Kyiv) [24]	Y	Y	Y	Low	Low	Y	Y	Low	Low	S+: Y	N	High	Low	Y	N	Y	High
Norway (nationwide) [25]	Y	Y	Y	Low	Low	Y	Y	Low	Low	S+: Y	N	High	Low	Y	N	Y	High
Australia (NSW + ACT) [26,27,28]	Y	Y	Y	Low	Low	Y	Y	Low	Low	S+: Y	N	High	Low	Y	N	Y	High
Australia (Queensland) [29]	Y	Y	Y	Low	Low	Y	Y	Low	Low	S+: Y	N	High	Low	Y	N	Y	High
Canada (Ontario) [30,31]	Y	Y	Y	Low	Low	Y	Y	Low	Low	S+: Y	N	High	Low	Y	N	Y	High
Canada (Alberta) [32]	Y	Y	Y	Low	Low	Y	Y	Low	Low	S+: Y	N	High	Low	Y	N	Y	High
USA (California) [33]	Y	Y	Y	Low	Low	Y	Y	Low	Low	S+: Y	N	High	Low	Y	N	Y	High
USA (Georgia State) [34]	Y	Y	Y	Low	Low	Y	Y	Low	Low	S+: Y	N	High	Low	Y	N	Y	High
USA (Kentucky) [35]	Y	Y	Y	Low	Low	Y	Y	Low	Low	S+: Y	N	High	Low	Y	N	Y	High
USA (Massachusetts) [36,37]	Y	Y	Y	Low	Low	Y	Y	Low	Low	S+: Y	N	High	Low	Y	N	Y	High
USA (New York State) [38,39]	Y	Y	Y	Low	Low	Y	Y	Low	Low	S+: Y	N	High	Low	Y	N	Y	High
USA (3 hospitals New York City) [40]	Y	Y	Y	Low	Low	Y	Y	Low	Low	S+: Y	N	High	Low	Y	N	Y	High
USA (North Carolina) [41]	Y	Y	Y	Low	Low	Y	Y	Low	Low	S+: Y	N	High	Low	Y	N	Y	High
USA (Wisconsin) [42]	Y	Y	Y	Low	Low	Y	Y	Low	Low	S+: Y	N	High	Low	Y	N	Y	High
USA (Utah) [43]	Y	Y	Y	Low	Low	Y	Y	Low	Low	S+: Y	N	High	Low	Y	N	Y	High
Brazil (Sao Paulo + Rio Grande) [45]	U	Y	U	Uncl	Low	Y	Y	Low	Low	S+: Y	N	High	Low	Y	N	Y	High
Japan (Kumamoto) [46]	Y	Y	Y	Low	Low	Y	Y	Low	Low	S+: Y	N	High	Low	Y	N	Y	High
Japan (Osaka) [47,48]	Y	Y	Y	Low	Low	Y	Y	Low	Low	S+: Y	N	High	Low	Y	N	Y	High
Japan (Hyogo) [49]	Y	Y	Y	Low	Low	Y	Y	Low	Low	S+: Y	N	High	Low	Y	N	Y	High
Japan (49 hosp, 23 prefectures) [50]	Y	Y	Y	Low	Low	Y	Y	Low	Low	S+: Y	N	High	Low	Y	N	Y	High
Taiwan (University Hospital) [51,52]	Y	Y	Y	Low	Low	Y	Y	Low	Low	S+: Y	N	High	Low	Y	N	Y	High
China (6 hospitals) [53]	Y	Y	Y	Low	Low	Y	Y	Low	Low	S+: Y	N	High	Low	Y	N	Y	High
Russia (Moscow) [56]	Y	Y	Y	Low	Low	Y	Y	Low	Low	S+: Y	N	High	Low	Y	N	Y	High
Russia (Saint Petersburg) [57]	Y	Y	Y	Low	Low	Y	Y	Low	Low	S+: Y	N	High	Low	Y	N	Y	High
Overviews of geographical areas																	
USA (Ohio) [44]	U	Y	U	Uncl	Low	Y	Y	Low	Low	Y	N	High	Low	Y	N	Y	High
China (Hunan province) [54]	Y	Y	Y	Low	Low	U	Y	Uncl	Low	U	N	High	Uncl	U	N	Y	High
China (southwest) [55]	Y	Y	Y	Low	Low	Y	Y	Low	Low	S+: Y	N	High	Low	Y	N	Y	High

Abbreviations: abst, abstract; N, no; Scr+, screen positives; Scr-, screen negatives; U, unclear; Y, yes. On each “risk of bias overall” criterion, studies scored Low if Y to all individual criteria, High if No to any criteria, and Unclear if some criteria were Unclear but none scored Low.

**Table 5:** Timing of testing process.

Study, Location	Median Time in Days (Range or Interquartile Range) from Birth to:						
	DBS Sampling	DBS Receipt	Initial Screening Results	Parent Contact	Specialist Consultation	Confirmatory Results	Start of Treatment
Belgium (southern) [10,11,12]	3 (3–4)	6 (4–13)	18 (9–31) 1st tier 21 (10–35) 2nd tier	20 (9–35)	21 (10–37)		38 (29–54)
Germany (Bavaria + NRW) [13,14,15,16,17]			6 (3–9)	7 (6–45)	8 (6–54)	13 (9–14)	19 (7–728)
Germany (nationwide) [17]			7 (4–15)	8 (4–15)	10 (5–46)	13 (9–19)	27 (13–66)
Italy (Lazio and Tuscany) [19]			6 (5–9)			11 (7–21)	17 (11–62)
Italy (Liguria) [20]						13	
Latvia [21]			11				
Poland [23]			9			15	
Norway [25]							NR (13–18)
Australia (NSW + ACT) [26,27,28]			3 (2–15)			15 (10–23)	25 (15–39)
Canada (Ontario) [30,31]	1	3 (3–6)	8 (5–13)	9 (6–15)	11 (9–16)	14 (12–24)	24 (18–32)
Canada (Alberta) [32]	1	2 (1–3)	7 (6–8)			15 (13–27)	29 (25–72)
USA (California) [33]			5 (1–10)		8 (5–15)	12 (3–27)	33 (17–79)
USA (Georgia state) [34]			5 (1–6)		33 (15–46)		106 (28–189)
USA (Kentucky) [35]			NR (2–13)				48 (16–331)
USA (Massachusetts) [36,37]	2 (1–2)		4 (3–6)		7 (0–26)		18 (8–171)
USA (New York State) [38,39]			7 (4–12)		9 (1–58)		35 (11–180)
USA (New York State pilot) [40]			3			5	15
USA (North Carolina) [41]						28 (19–36)	30
USA (Wisconsin) [42]	1 (1–2)		3 (3–6)				19 (11–57)
Brazil [45]	6 (4–60)	75 (45–90)					
Japan (Kumamoto) [46]	5		13			19	42
Japan (Osaka) [47,48]	NR (4–6)	6 (4–15)	NR (6–13)		NR (7–18)	NR (10–28)	21, 29
Japan (Hyogo) [49]	NR (4–6)					19, 23	22, 25
Russia (Moscow) [56]	4	NR (4–6)	NR (6–8)				

Abbreviations: ACT, Australian Capital Territory; DBS, dried blood spot; IQR, interquartile range; NR, not reported; NRW, North Rhine-Westphalia; NSW, New South Wales.

**Table 6:** Workflow and consent.

**Study, Location**	**Workflow**
Belgium (southern) [10,11,12]	Samples analysed per week: 300–350 (in first 9 months); 1200 (after expansion)
Germany (Bavaria + NRW) [13,14,15,16,17]	Aimed to screen up to 2000 samples per day with one person operating the molecular genetic screening procedure
Germany (Heidelberg) [18]	On peak days, >1000 samples could be processed for multiplex qPCR
Latvia [21]	83 samples analysed in first month; 1054 analysed in final month
Australia (Queensland) [29]	Laboratory and bioinformatics software automation procedures developed, to screen over 200 samples per day. Weekly batch size of 1536 samples
USA (Ohio) [44] (anonymised DBS)	Utilising two instruments and two technologists enabled assay on 400–500 samples daily
**Study, Location**	**Consent processes**
Global overview [58]	Some countries use opt-in (Germany, Italy, Japan, Taiwan, Russia) and some opt-out (USA, Canada, Belgium, Australia)
Canada overview [59]	Most provinces screen for SMA alongside other newborn screening and do not require specific consent, while Alberta has an opt-out process
UK (Thames Valley) [9]	Initial uptake of antenatal consent was slow with staff availability the main limiting factor. Consent rate increased with remote consenting and with postnatal consent during baby checks
Italy (Lazio and Tuscany) [19]	Consent of families: 91% during pilot, 98–99% when routine screening started
Italy (Liguria) [20]	Consent rate 99.9%
Latvia [21]	Consent rate approximately 70%
USA (New York City pilot) [40]	Consent rate 93%
Japan (Osaka) [47,48]	Consent rate 98%
Russia (Moscow) [56]	No parents declined participation
Russia (Saint Petersburg) [57]	Consent rate 99.8%

**Table 7:** Implementation and barriers.

Study, Location	Implementation and Barriers
Global overview [58]	Implementation considerations: Start with pilot project Identify process for implementation (screening assay, staffing, funding, regulatory requirements, speciality referral centres) Educate colleagues and policy makers about presymptomatic treatment initiation Present long-term efficacy of treatment Share experience of implementation process Use a whole health systems approach and partner with patient organisations Barriers and uncertainties: Cost-effectiveness issues and reimbursement of treatment Uncertainties about management of patients with ≥4 *SMN2* copies Carrier testing
Germany (nationwide) [17]	Implementation considerations: Process of converting from pilot to nationwide screening required consideration of the following: Selection of specialist centres Criteria for follow-up care Developing information for laboratories, clinics and parents Barriers: In 4 cases, uncertainty about which neurological centre should provide care
Australia (NSW + ACT) [26,27,28]	Implementation considerations: Screening pathways reviewed to avoid delays in referral and diagnosis of screen positives Root cause analysis of false-negatives Confirmation of *SMN1* deletion in new blood sample with different primers Flexibility of team to change work patterns to deal with urgent cases Set up system for rapid *SMN2* testing (lower *SMN2* copy number cases were then triaged faster) Strong partnerships between newborn screening staff and neuromuscular specialists Tailored information to fit a variety of needs among families Focus on family-centred care: before contacting families, identified most appropriate clinical setting for consultation; options included immediate referral to neuromuscular team, or local consultation with specialist tele-health support if difficulties travelling long distances Genetic counselling, family cascade testing, psychosocial support If presymptomatic, uncertainty in conversations about clinical severity and long-term outcomes (reliant on *SMN2* copy number) Changing access to presymptomatic disease-modifying therapies; also limited access for 3+ *SMN2* copies
Canada overview [59]	Barriers: Most Canadian provinces require a positive confirmatory genetic test prior to application for treatment, which can result in an additional 1–2 week delay in initiating treatment, while Saskatchewan allows application after a positive initial screen
Canada overview [59]	Implementation considerations: Modifications that could potentially reduce time to treatment initiation: Operation of newborn screening laboratory on weekends Reduction in time to transport sample from the collection site to newborn screening laboratory Reduction of time required for confirmatory testing Submission of preliminary paperwork for provincial Exceptional Access Program approval while awaiting the results of the confirmatory genetic testing
USA (California) [33]	Barriers: Half (9/18) infants had treatment in a timely manner. Most common barriers or reasons for delay to treatment: Problems with insurance authorisation (*n* = 6) Logistical issues getting patient to centre for treatment (*n* = 2) Delays due to SMA-related health issues (*n* = 1) or other health issues (*n* = 2) Delays in receiving confirmatory results (*n* = 2) Delays at the pharmacy (*n* = 1) For 2 cases, clinicians noted delays may have been compounded by the new process for newborn screening
USA (Kentucky) [35]	Barriers: Factors causing delayed treatment: Insurance denial (*n* = 4) Abnormal lab results (*n* = 3) Prematurity (*n* = 1)
USA (New York State) [38,39]	Barriers: Medical delays most commonly reported were the presence of AAV9 antibodies and elevated troponin I levels Nonmedical barriers included delays in obtaining insurance and insurance policies regarding specific treatment modalities
Japan (Osaka) [47,48]	Barriers: Some samples were delayed in arriving at the lab, mainly due to problems with transportation over weekends and public holidays
Russia (Moscow) [56]	Barriers: Logistical issues: Inconsistency between number of samples of newborns in database and number of forms delivered to lab Depersonalisation of samples led to problems with summoning families for validation of positive results

Abbreviations: ACT, Australian Capital Territory; NSW, New South Wales; SMA, spinal muscular atrophy; *SMN1/2*, survival motor neuron 1/2.

## Appendix A. Medline Search Strategy

1. exp “Spinal Muscular Atrophies of Childhood”/
2. exp Muscular Atrophy, Spinal/
3. (werdnig-hoffman or werdnig hoffman).tw.
4. (kugelberg-welander or kugelberg welander).tw.
5. spinal muscular atroph*.tw.
6. or/1–5
7. exp Neonatal Screening/
8. ((neonat* or newborn?) adj2 (screen* or detect* or diagnos* or test*)).ti,ab.
9. 7 or 8
10. 6 and 9

## Appendix B. List of Case-Control Studies of Newborn Screening for SMA

The following case-control studies of newborn screening for SMA were identified in the systematic review conducted by SCHARR via searches of MEDLINE, Embase and the Cochrane Library; searches were conducted in November 2023 and covered all dates up to this point.

**Table A1:** Case-control studies of newborn screening for SMA.

Country (State/Area)	Reference	Also Reports Cohort Study	Full Reference
UK	Adams 2021		Adams SP, Gravett E, Kent N, et al. Screening of Neonatal UK Dried Blood Spots Using a Duplex SMN1 Screening Assay. *International Journal of Neonatal Screening* 2021;7:26. doi:10.3390/ijns7040069
Belgium	Boemer 2019	Y	Boemer F, Caberg JH, Dideberg V, et al. Newborn screening for SMA in Southern Belgium. *Neuromuscular Disorders* 2019;29(5):343–9. doi:10.1016/j.nmd.2019.02.003
Germany (Bavaria)	Czibere 2020	Y	Czibere L, Burggraf S, Fleige T, et al. High-throughput genetic newborn screening for spinal muscular atrophy by rapid nucleic acid extraction from dried blood spots and 384-well qPCR. *European Journal of Human Genetics* 2020;28:23–30. doi:10.1038/s41431-019-0476-4
Germany (Heidelberg)	Tesorero 2023	Y	Tesorero, R., J. Janda, F. Horster, P. et al. A High-Throughput Newborn Screening Approach for SCID, SMA, and SCD Combining Multiplex QPCR and Tandem Mass Spectrometry. *PLoS ONE* 18, no. 3 (2023): e0283024.
Denmark	Gutierrez-Mateo 2019		Gutierrez-Mateo C, Timonen A, Vaahtera K, et al. Development of a Multiplex Real-Time PCR Assay for the Newborn Screening of SCID, SMA, and XLA. *International Journal of Neonatal Screening* 2019;5:39. doi:10.3390/ijns5040039.
Netherlands	Strunk 2019		Strunk A, Abbes A, Stuitje AR, et al. Validation of a Fast, Robust, Inexpensive, Two-Tiered Neonatal Screening Test algorithm on Dried Blood Spots for Spinal Muscular Atrophy. *International Journal of Neonatal Screening* 2019;5:21. doi:10.3390/ijns5020021.
Turkey	Cavdarli 2020		Cavdarli B, Ozturk FN, Guntekin Ergun S, et al. Intelligent Ratio: A New Method for Carrier and Newborn Screening in Spinal Muscular Atrophy. *Genetic Testing & Molecular Biomarkers* 2020;24:569–77. doi:10.1089/gtmb.2020.0085
Australia (Queensland)	Shum 2023	Y	Shum BOV, Henner I, cairns A et al. Technical feasibility of newborn screening for spinal muscular atrophy by next-generation DNA sequencing. *Frontiers in Genetics* 2023;14.
Canada (Alberta)	Niri 2023	Y	Niri, F., J. Nicholls, K. Baptista Wyatt, C., et al. Alberta Spinal Muscular Atrophy Newborn Screening-Results from Year 1 Pilot Project. *International Journal of Neonatal Screening* 9, no. 3 (2023): 27.
USA (New York State)	Kraszewski 2018	Y	Kraszewski JN, Kay DM, Stevens CF, et al. Pilot study of population-based newborn screening for spinal muscular atrophy in New York state. *Genetics in Medicine* 2018;20:608–13. doi:10.1038/gim.2017.152
USA (Ohio)	Pyatt 2007		Pyatt RE, Mihal DC, Prior TW. Assessment of liquid microbead arrays for the screening of newborns for spinal muscular atrophy. *Clinical Chemistry* 2007;53:1879–85.
USA (Ohio)	Pyatt 2006		Pyatt RE, Prior TW. A feasibility study for the newborn screening of spinal muscular atrophy. *Genetics in Medicine* 2006;8:428–37.
Turkey	Kubar 2023		Kubar A, Gülsüm Temel S, Beken S et al. A new line method; A direct test in spinal muscular atrophy screening for DBS. *Molecular Genetics & Genomic Medicine* 2023;0:e2270.
USA (North Carolina)	Kucera 2021	Y	Kucera KS, Taylor JL, Robles VR, et al. A Voluntary Statewide Newborn Screening Pilot for Spinal Muscular Atrophy: Results from Early Check. *International Journal of Neonatal Screening* 2021;7:21. doi:10.3390/ijns7010020
USA (North Carolina)	Taylor 2015		Taylor JL, Lee FK, Yazdanpanah GK, et al. Newborn blood spot screening test using multiplexed real-time PCR to simultaneously screen for spinal muscular atrophy and severe combined immunodeficiency. *Clinical Chemistry* 2015;61:412–9. doi:10.1373/clinchem.2014.231019
USA	Vidal-Folch 2018		Vidal-Folch N, Gavrilov D, Raymond K, et al. Multiplex Droplet Digital PCR Method Applicable to Newborn Screening, Carrier Status, and Assessment of Spinal Muscular Atrophy. *Clinical Chemistry* 2018;64:1753–61. doi:10.1373/clinchem.2018.293712
Brazil	Romanelli Tavares 2021		Romanelli Tavares VL, Monfardini F, Lourenco NCV, et al. Newborn Screening for 5q Spinal Muscular Atrophy: Comparisons between Real-Time PCR Methodologies and Cost Estimations for Future Implementation Programs. *International Journal of Neonatal Screening* 2021;7:11. doi:10.3390/ijns7030053
Brazil	Silva 2023 (abstract)		Silva, Jd, da Silva CM, Zauli DA et al. Molecular Assay Evaluation to SMA and SCID Diagnosis in Newborn Dried Blood Spots (DBS). *Clinical Chemistry* 2023;69:i236-i237.
Japan	Ar Rochmah 2017		Ar Rochmah M, Harahap NIF, Niba ETE, et al. Genetic screening of spinal muscular atrophy using a real-time modified COP-PCR technique with dried blood-spot DNA. *Brain & Development* 2017;39:774–82. doi:10.1016/j.braindev.2017.04.015
Japan (Osaka)	Kimizu 2021	Y	Kimizu T, Ida S, Okamoto K, et al. Spinal Muscular Atrophy: Diagnosis, Incidence, and Newborn Screening in Japan. *International Journal of Neonatal Screening* 2021;7:20. doi:10.3390/ijns7030045
Japan (all)	Shinohara 2019	Y	Shinohara M, Niba ETE, Wijaya YOS, et al. A Novel System for Spinal Muscular Atrophy Screening in Newborns: Japanese Pilot Study. *International Journal of Neonatal Screening* 2019;5:41. doi:10.3390/ijns5040041
Japan	Wijaya 2021		Wijaya YOS, Nishio H, Niba ETE, et al. Dried Blood Spot Screening System for Spinal Muscular Atrophy with Allele-Specific Polymerase Chain Reaction and Melting Peak Analysis. *Genetic Testing & Molecular Biomarkers* 2021;25:293–301. doi:10.1089/gtmb.2020.0312
Japan	Wijaya 2021		Wijaya YOS, Nishio H, Niba ETE, et al. Detection of Spinal Muscular Atrophy Patients Using Dried Saliva Spots. *Genes* 2021;12:14. doi:10.3390/genes12101621
Taiwan	Chien 2017	Y	Chien YH, Chiang SC, Weng WC, et al. Presymptomatic Diagnosis of Spinal Muscular Atrophy Through Newborn Screening. *Journal of Pediatrics* 2017;190:124-129.e1. doi:10.1016/j.jpeds.2017.06.042
Taiwan	Er 2012		Er T-K, Kan T-M, Su Y-F, et al. High-resolution melting (HRM) analysis as a feasible method for detecting spinal muscular atrophy via dried blood spots. *Clinica Chimica Acta* 2012;413:1781–5. doi:10.1016/j.cca.2012.06.033
Taiwan	Wang 2021		Wang KC, Fang CY, Chang CC, et al. A rapid molecular diagnostic method for spinal muscular atrophy. *Journal of Neurogenetics* 2021;35:29–32. doi:10.1080/01677063.2020.1853721
China	Lin 2019	Y	Lin Y, Lin CH, Yin X, et al. Newborn Screening for Spinal Muscular Atrophy in China Using DNA Mass Spectrometry. *Frontiers in Genetics* 2019;10:1255. doi:10.3389/fgene.2019.01255
China	Liu 2016	Y	Liu Z, Zhang P, He X, et al. New multiplex real-time PCR approach to detect gene mutations for spinal muscular atrophy. *BMC Neurology* 2016;16:141. doi:10.1186/s12883-016-0651-y
China	Pan 2021	Y	Pan J, Zhang C, Teng Y, et al. Detection of Spinal Muscular Atrophy Using a Duplexed Real-Time PCR Approach With Locked Nucleic Acid-Modified Primers. *Annals of Laboratory Medicine* 2021;41:101–7. doi:10.3343/alm.2021.41.1.101
Russia	Kiselev 2024	Y	Kiselev A, Maretina M, Shtykalova S, et al. Establishment of a Pilot Newborn Screening Program for Spinal Muscular Atrophy in Saint Petersburg. *IJNS*. 2024;10:9. doi: 10.3390/ijns10010009
Russia	Nazarov 2023		Nazarov VD, Cherebillo CC, Lapin SV et al. Detection of SMN1 loss with PCR-based screening test. Bulletin of Russian State Medical University 2023; 0(3):21-27.
Unclear	Guo 2021 (abstract)		Guo F, Ou Y, Mathur A, et al. Reducing the time to diagnosis for spinal muscular atrophy. *Molecular Genetics and Metabolism* 2021;132(Supplement 1):S279. doi:10.1016/S1096-7192%2821%2900513-8
